# The first isolate of *Candida auris* in China: clinical and biological aspects

**DOI:** 10.1038/s41426-018-0095-0

**Published:** 2018-05-18

**Authors:** Xiaojuan Wang, Jian Bing, Qiushi Zheng, Feifei Zhang, Jingbo Liu, Huizhen Yue, Li Tao, Han Du, Yina Wang, Hui Wang, Guanghua Huang

**Affiliations:** 1 Department of Clinical Laboratory, Peking University People’s Hospital, 100044 Beijing, China; 2 State Key Laboratory of Mycology, Institute of Microbiology, Chinese Academy of Sciences, 100101 Beijing, China; 3 University of Chinese Academy of Sciences, 100049 Beijing, China; 4 State Key Laboratory of Genetic Engineering, School of Life Sciences, Fudan University, 200438 Shanghai, China; 5 Department of Nephrology, Peking University People’s Hospital, 100044 Beijing, China

## Abstract

The emerging human fungal pathogen *Candida auris* has been recognized as a multidrug resistant species and is associated with high mortality. This fungus was first described in Japan in 2009 and has been reported in at least 18 countries on five continents. In this study, we report the first isolate of *C*. *auris* from the bronchoalveolar lavage fluid (BALF) of a hospitalized woman in China. Interestingly, this isolate is susceptible to all tested antifungals including amphotericin B, fluconazole, and caspofungin. Copper sulfate (CuSO_4_) also has a potent inhibitory effect on the growth of this fungus. Under different culture conditions, *C*. *auris* exhibits multiple morphological phenotypes including round-to-ovoid, elongated, and pseudohyphal-like forms. High concentrations of sodium chloride induce the formation of a pseudohyphal-like form. We further demonstrate that *C. auris* is much less virulent than *Candida albicans* in both mouse systemic and invertebrate *Galleria mellonella* models.

## Introduction

The incidence of non-albicans *Candida* infections has risen dramatically in recent years^1^. These species are often drug-resistant and difficult to eradicate from the human body, as well as the hospital environment. *Candida auris*, an emerging fungal pathogen of humans, is often resistant to multiple currently used drugs^2–4^. Fungemia caused by *C. auris* is associated with a high mortality rate and therapeutic failure^5–7^. Since the first report of *C. auris* infection in Japan in 2009, this fungus has been isolated on five continents^2,4^. In a national survey of intensive care units (ICUs), *C. auris* was reported to account for >5% of candidemia in India^5,8^. The ecological niches for this fungus remain unidentified. However, their survival and persistence ability on dry surfaces and within hospital environments may contribute to the prevalence and outbreaks of *C. auris* worldwide.

The morphological diversity and secreted aspartyl proteinases (Saps) are important virulence features of pathogenic *Candida* species^9–12^. For example, *Candida albicans*, the major fungal pathogen of humans, has multiple morphological cell types, including the yeast form, hyphae, pseudohyphae, and white, gray, and opaque cell types^9,13^. *C. albicans* can switch among different morphologies under certain culture conditions or during infection^13^. Moreover, it has 10 genes encoding Saps that are the major virulence factors responsible for host tissue damage^10^.

Although *C*. *auris* has attracted great attention in the clinical and scientific fields and more than 80 related research and review papers have been published in the past several years, knowledge of the biology and virulence features of this organism is still limited. In this study, we report the first isolate of *C*. *auris* in China. We further investigate its morphological characteristics under different culture conditions and its virulence in both mouse and invertebrate *Galleria mellonella* models.

## Results

### The first isolate of *C*. *auris* in China and analysis of its antifungal susceptibility

**Fig. 1:**
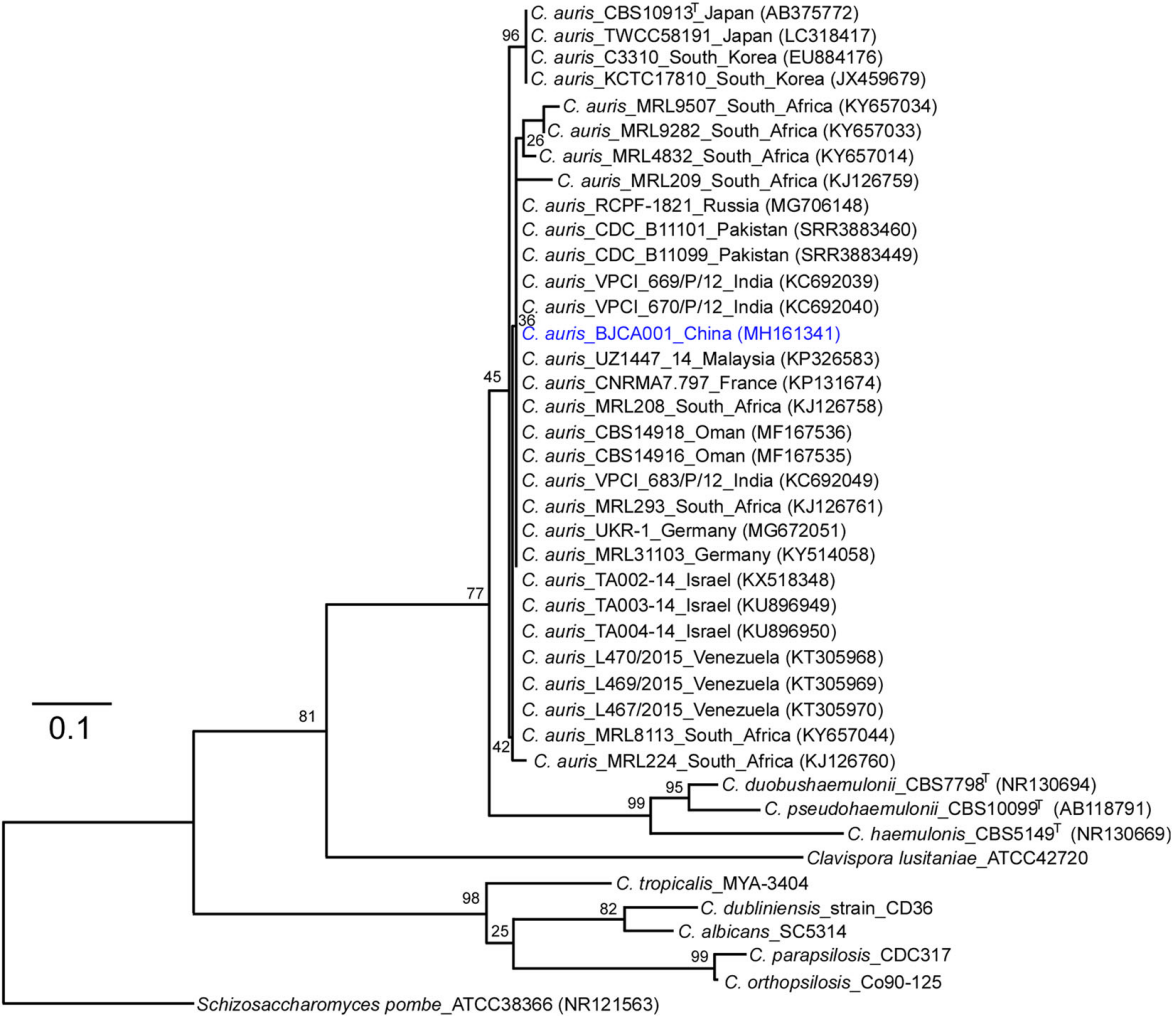
Phylogenetic trees generated by the Maximum- Likelihood (ML) method. Internal transcribed spacer (ITS) sequences of nuclear rDNA of *Candida auris*, *C*. *auris* closely related species, and *Schizosaccharomyces pombe* were used. The GenBank accession numbers are shown in the brackets. *Candida* species used: *C*. *duobushaemulonii, C*. *pseudohaemulonii, C*. *haemulonis, C*. *tropicalis, C*. *dubliniensis, C*. *albicans, C*. *parapsilosis*, and *C*. *orthopsilosis*. The Maximum-Likelihood phylogenetic tree was generated using RAxML based on the General Time Reversible (GTR) model and Gamma distribution with Invariant sites (G + I). The percentages of replicate trees in which the associated taxa clustered together in the bootstrap test (1000 replicates) are indicated at the branches. The scale bar indicates the nucleotide substitutions per site. Strain BJCA001 is highlighted in blue

A 76-year-old woman with hypertension and nephritic syndrome was admitted to Peking University People’s Hospital. An isolate of *C*. *auris* (BJCA001) was identified from the bronchoalveolar lavage fluid (BALF). We did not isolate *C*. *auris* cells from the hospital facility and the other parts of the patient’s body. The strain was initially identified as *C*. *auris* by applying in-house MALDI-TOF MS database (with confidence > 1.8). Genomic DNA was extracted for further verification by molecular identification methods. The sequence of the internal transcribed spacers (ITS) of BJCA001 showed 99.9% identity to those of several reported *C*. *auris* isolates^3,8^. To further verify this finding, we sequenced a *C*. *auris*-specific ORF (XM_018314828.1) in strain BJCA001. We then performed a phylogenetic analysis using ITS sequences (Fig. 1). To our surprise, strain BJCA001 had very low minimal inhibitory concentration (MIC) values of all the tested drugs (Table 1). The MICs of amphotericin B, fluconazole, and anidulafungin were 0.25, 2.0, and 0.12 μg/mL, respectively, whereas the MICs of flucytosine, itraconazole, caspofungin, micafungin, posaconazole, and voriconazole were less than 0.1 μg/mL.

**Table 1:** Antifungal susceptibility testing of *Candida auris* BJCA001

	FLC	ITC	POS	VRC	AMB	5-FC	AFG	CAS	MFG
MIC (µg/mL)	2	0.03	0.02	0.02	0.25	< 0.06	0.12	0.06	0.06

*FLC* fluconazole, *ITC* itraconazole, *POS* posaconazole, *VRC* voriconazole, *AMB* amphotericin B, *5-FC* 5-flucytosine, *AFG* anidulafungin, *CAS* caspofungin, *MFG* micafungin, *MIC* minimal inhibitory concentration (μg/mL)

### Inhibitory effect of copper sulfate (CuSO_4_) on *C*. *auris*

**Fig. 2:**
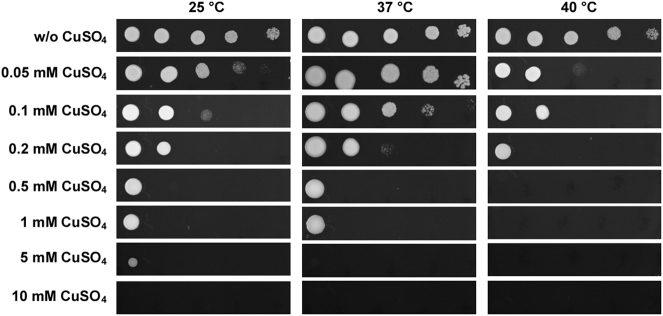
Inhibitory effect of CuSO_4_ on the growth of *C*. *auris* grown at 25 °C, 37 °C, and 40 °C. *C*. *auris* cells were adjusted to 5 × 10^8^ cells/mL, and then 10-fold serial dilutions of cells (2 μL) were spotted onto YPD and YPD containing CuSO_4_ media for four days of growth

Copper is a potent antimicrobial agent^14,15^ and copper-based compounds have long been used as biocontrol agents. Therefore, we examined whether CuSO_4_ had antifungal activity toward *C*. *auris*. As shown in Fig. 2, serial dilution assays demonstrated that CuSO_4_ exhibited a potent inhibitory effect on the cell growth of *C*. *auris*, especially at high temperatures (>37 °C). At 40 °C, 0.5 mM of CuSO_4_ completely inhibited the growth of *C*. *auris* on YPD medium, whereas no growth was observed in the presence of 5 mM and 10 mM of CuSO_4_ at 37 °C and 25 °C, respectively.

### Morphological analysis of *C*. *auris*

**Fig. 3:**
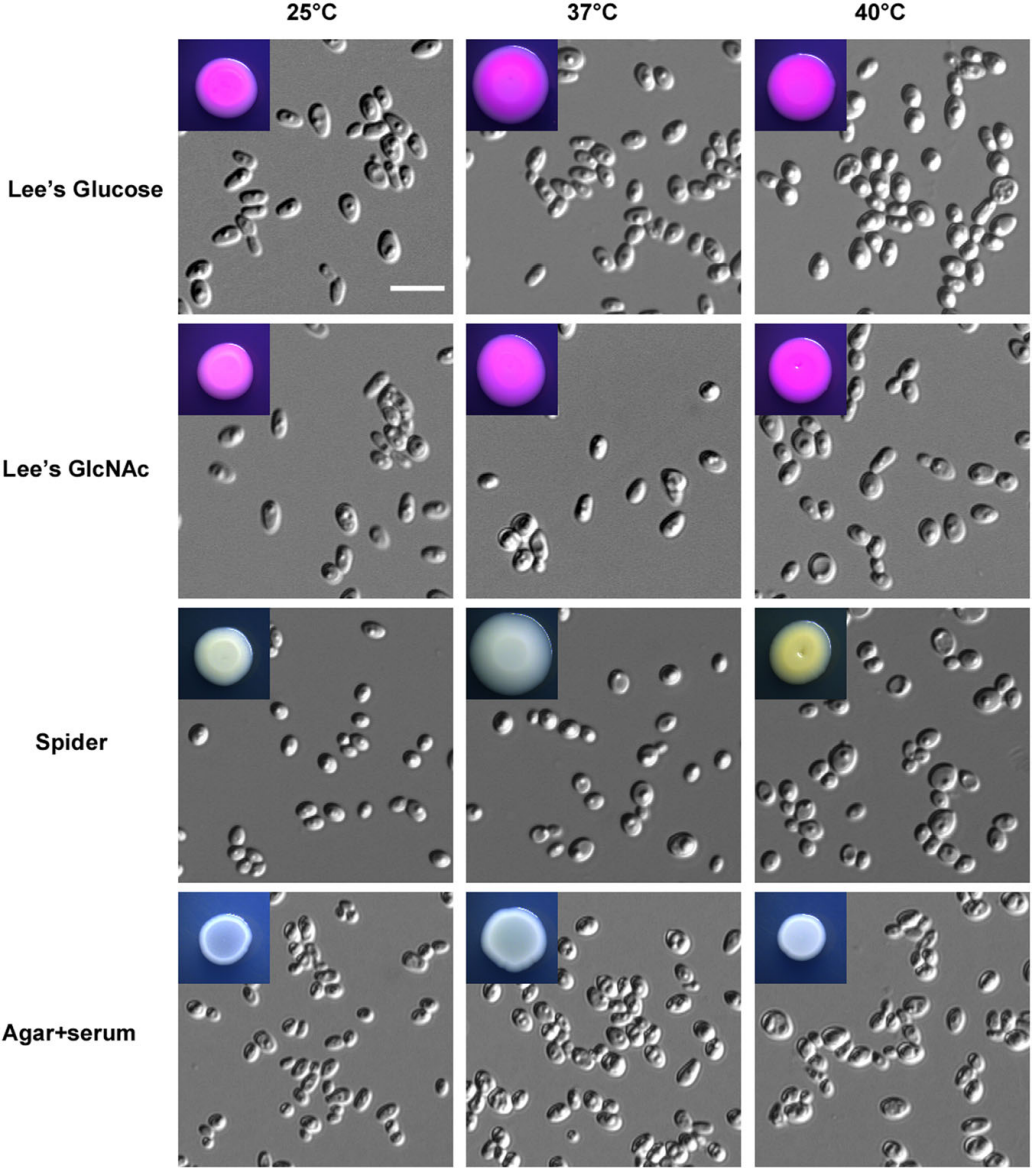
Morphologies of *C*. *auris* grown on Lee’s glucose, Lee’s GlcNAc, Spider, and agar + serum media. Cells (1 × 10^5^) were spotted onto different medias and cultured at 25 °C, 37 °C, and 40 °C for five days. Scale bar, 10 μm

Morphological diversity is a key virulence feature of *Candida* species^9,12,16^. To investigate whether *C*. *auris* had multiple cellular morphologies, we cultured cells using several growth media at 25 °C, 37 °C, and 40 °C (Fig. 3). On Lee’s glucose and Lee’s GlcNAc media, *C*. *auris* cells exhibited an oval shape at 25 °C and 37 °C and a relative round shape at 40 °C. On Spider and agar plus serum media, cells were round and relatively small. No hyphal and pseudohyphal cells were observed under these conditions.

**Fig. 4:**
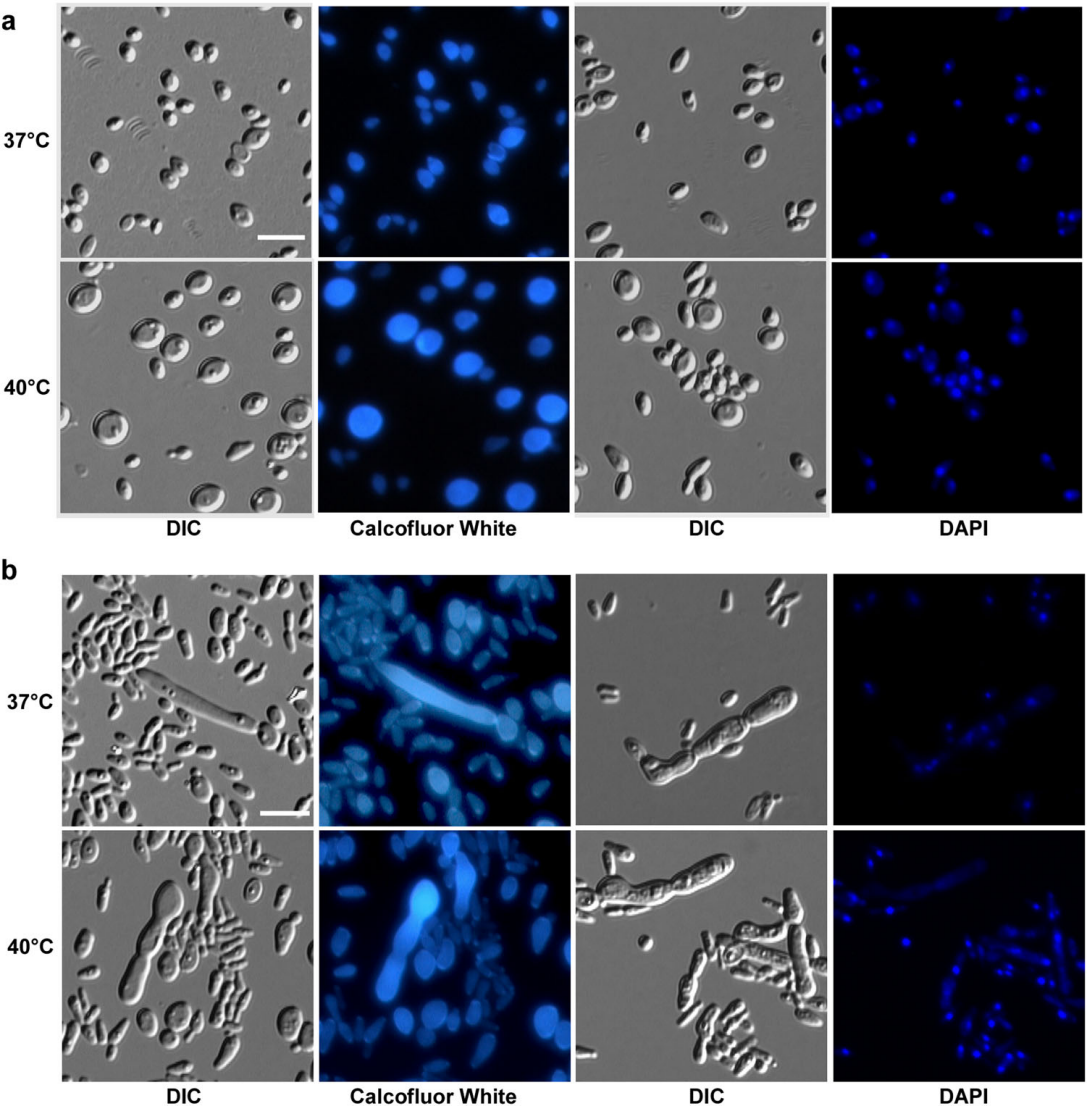
Morphologies of *C*. *auris* cells grown on YPD (**a**) and YPD plus 10% NaCl (**b**) media. Cells (1 × 10^5^) were spotted onto different medias and cultured at 37 °C and 40 °C for five days. Cells were collected and stained with DAPI or Calcofluor white. Scale bar, 10 μm. DIC, differential interference contrast

**Fig. 5:**
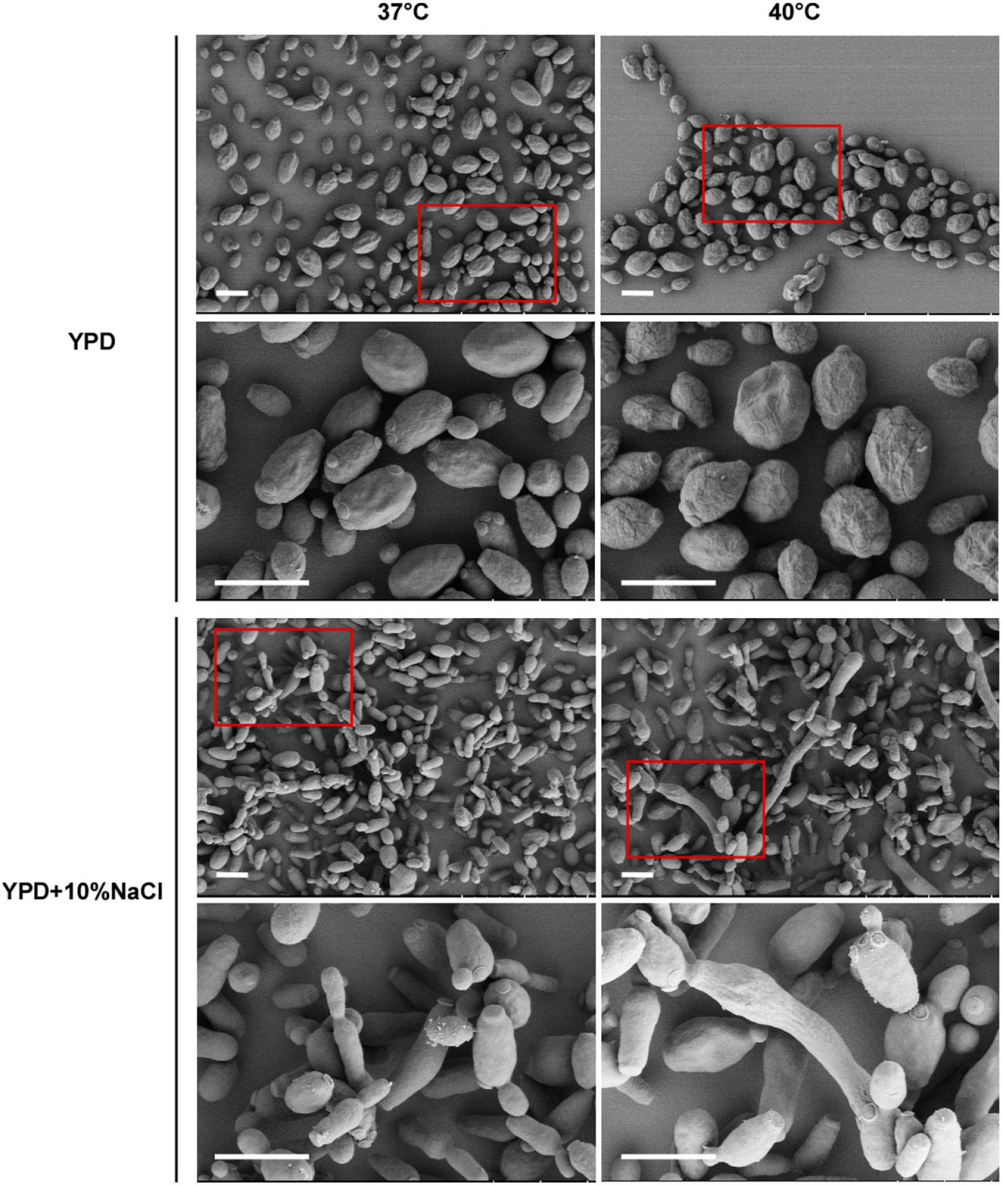
Scanning electron microscopy images (SEM) of *C*. *auris* cells grown on YPD and YPD plus 10% NaCl media. Cells (1 × 10^5^) were spotted onto different media and cultured at 37 °C and 40 °C for five days. Cells were then collected for SEM assays. Scale bar, 5 μm

Since it has been reported that *C*. *auris* can grow at high salt concentrations and temperatures^17^, we next examined the morphology of *C*. *auris* on the rich medium YPD and YPD plus 10% NaCl. As shown in Figs. 4 and 5, *C*. *auris* cells were round on regular YPD medium, but they showed an elongated shape on YPD plus 10% NaCl. Elongated cells of *C*. *auris* resembled opaque cells of *C*. *albicans* in shape^18^. Interestingly, we observed a small portion of highly elongated and pseudohyphal-like cells when they were grown on YPD plus 10% NaCl. Multiple nuclei were observed in the elongated cells by staining with DAPI. However, no septin/chitin rings were observed between conjoint cells when stained with Calcofluor white (Fig. 4). These results suggest that the high-salt stress could lead to incomplete cell division and the formation of pseudohyphal-like cells.

### Sap activity of *C*. *auris* at different temperatures

**Fig. 6:**
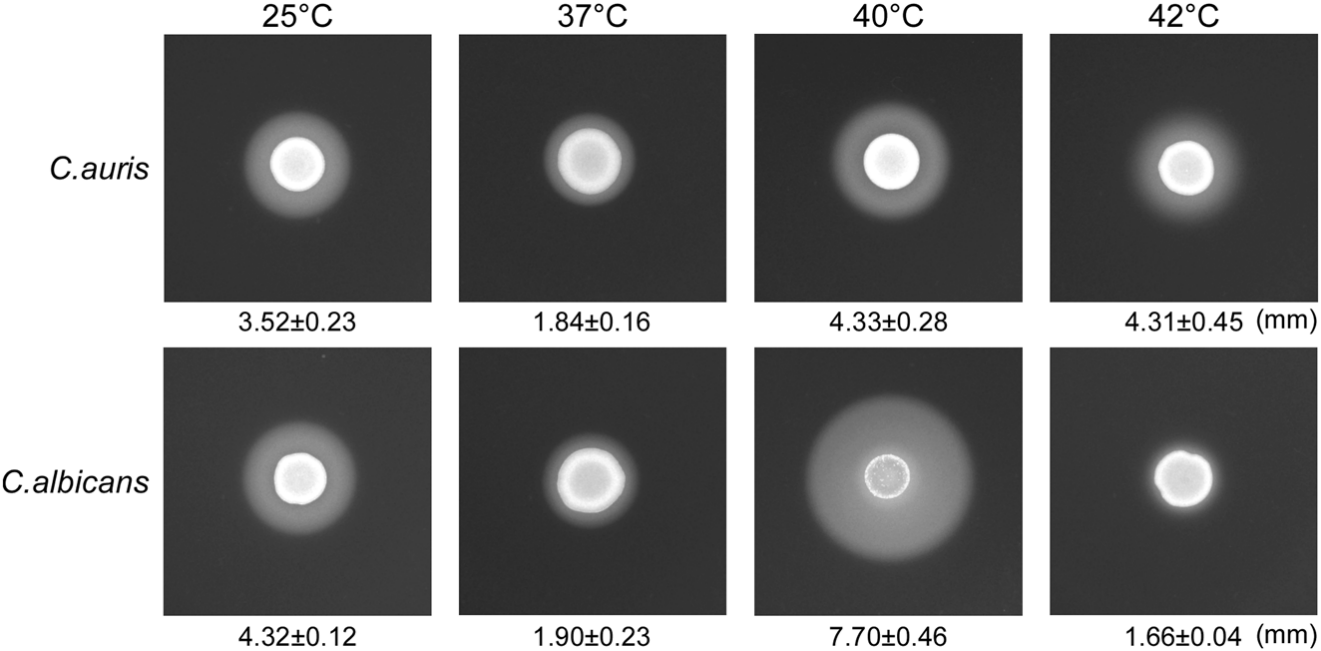
A comparison of Sap activities of *C*. *auris* and *C*. *albicans*. We spotted 5 × 10^6^ cells of *C*. *auris* or *C*. *albicans* (SC5314) in 5 µL ddH_2_O onto YCB-BSA medium plates, followed by growth at 25 °C, 37 °C, 40 °C, and 42 °C for five days. The white precipitation zones (halos) around the cell spots indicate Sap-mediated BSA hydrolysis. The width of the precipitation zones is indicated below the corresponding image. Average values of three biological repeats and standard deviations are presented

Secreted aspartyl proteinase (Saps) are important virulence factors that cause host tissue damages in *Candida* species^10^. To uncover this virulence feature, we examined Sap activity in *C*. *auris* using YCB-BSA assays. As shown in Fig. 6, *C*. *auris* exhibited high Sap activity at 25 °C, 37 °C, 40 °C, and even 42 °C. *C*. *albicans* showed high Sap activity at 25 °C, 37 °C, and 40 °C, but showed significantly reduced Sap activity at 42 °C.

### Virulence of *C*. *auris* in mouse systemic infection models

**Fig. 7:**
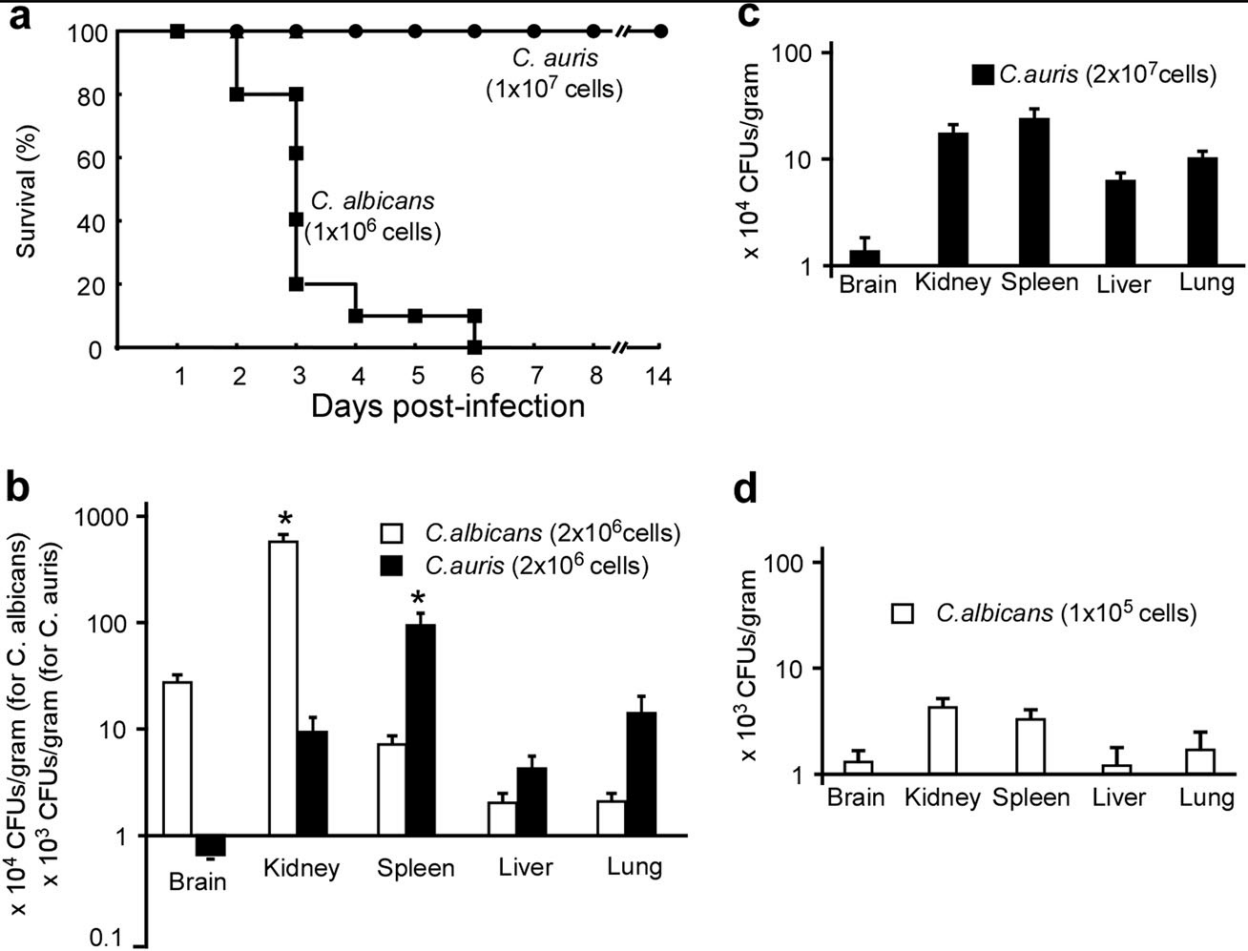
Virulence of *C*. *auris* and *C*. *albicans* in mouse systemic infection models. **a** Survival curves of mice injected with *C*. *auris* (1 × 10^7^ cells/mouse) and *C*. *albicans* (1 × 10^6^ cells/mouse) via the lateral tail vein. Ten mice were used for each strain. **b–d** Fungal burden assays. Five mice were used for each infection group. Mice were killed for CFU assays at 24 h post-infection. **b** Each mouse was injected with 2 × 10^6^ cells of *C*. *auris* or *C*. *albicans*. **c** Each mouse was injected with 2 × 10^7^ cells of *C*. *auris*. **d**, Each mouse was injected with 1 × 10^5^ cells of *C*. *albicans*. * Indicates a significant difference (*P* vale < 0.01, *Student’s t*-test, two-tailed) compared with the fungal burden in other organs

To evaluate the infectious ability of *C*. *auris*, we performed both survival and fungal burden assays using mouse systemic infection models. *C*. *albicans* (SC5314) was used as a control strain. As shown in Fig. 7a, all mice died at the sixth day post infection when injected with 1 × 10^6^ *C*. *albicans* cells per mouse via the vein tail. However, no mice died even at 14 days post-infection after injection 1 × 10^7^ *C*. *auris* cells per mouse. Consistent with a previous study^19^, our results suggest that *C*. *auris* is much less virulent than *C*. *albicans*.

We further found that the fungal burden in kidney, spleen, lung, and liver were comparable when 2 × 10^7 ^*C*. *auris* cells were injected into each mouse, while fungal burden in the spleen was significantly higher than that in the kidney, lung, and liver when 2 × 10^6 ^*C*. *auris* cells were injected (Fig. 7b, c). For *C*. *albicans*, the fungal burden in the kidney was significantly higher than those in the spleen, lung, and liver following injection of 2 × 10^6^ cells. However, the fungal burden in the kidney and spleen were comparable following injection of 2 × 10^5 ^*C*. *albicans* cells (Fig. 7d).

### Virulence of *C*. *auris* in a *G*. *mellonella* infection model

To further characterize the virulence features of *C*. *auris*, we next performed infection assays using a *G*. *mellonella* model. As shown in Figure S1a and S1b, *C*. *auris* exhibited reduced virulence compared with *C*. *albicans* when 2 × 10^5^ cells were injected into each *G*. *mellonella* larva, although the two fungi resulted in comparable survival rates following injection of 1 × 10^6^ cells were injected. Interestingly, *C*. *glabrata* was much less virulent than *C*. *auris* and *C*. *albicans* in the *G*. *mellonella* infection model (Figure S1c).

## Discussion

The novel fungal species, *C*. *auris*, is becoming a serious threat to global health. Since its first description in Japan in 2009^2^, *C*. *auris* infections have been reported in at least 18 countries^4^. In the present study, we report the first isolate of *C*. *auris* from the bronchoalveolar lavage fluid of a hospitalized woman in Beijing, China. This fungus had not been isolated in China previously perhaps due to technical reasons. *C*. *auris* is often misidentified as *C*. *haemulonii* using conventional methods^20^. We further investigated the morphological phenotypes, susceptibility to antifungal agents and CuSO_4_, and virulence of *C*. *auris* using both mouse and *G*. *mellonella* infection models.

Previous investigations have demonstrated multiple geographic origins of C. *auris* infections^7^. Although most of the previously reported *C*. *auris* strains exhibit multidrug resistance, to our surprise, BJCA001 was susceptible to all the tested antifungals (Table 1). Therefore, it is unknown whether the drug resistance of previously reported *C*. *auris* isolates is a recently evolved feature or whether strain BJCA001 has lost antifungal resistance.

*C*. *auris* is difficult to eradicate from the hospital environment due to its ability to survive on surfaces^21^. Some *C*. *auris* strains can even tolerate sanitizers such as sodium hypochlorite and peracetic acid^21^. Our discovery of its susceptibility to CuSO_4_ may provide a new avenue to eradicate this organism from the hospital environment (Fig. 2).

Consistent with a previous study^19^, we did not observe hyphal growth of *C*. *auris* under a variety of culture conditions. However, it did exhibit multiple cellular morphologies including round, elongated, and pseudohyphal-like forms (Figs. 3–5). High concentrations of NaCl induce the development of elongated and pseudohyphal-like cells in *C*. *auris* (Figs. 4 and 5). The mechanism of this induction and the roles of different cell types during infection remain to be investigated. It is unknown whether the elongated cell type of *C*. *auris* is similar to the opaque phenotype of *C*. *albicans*^18^.

Virulence assays demonstrated that *C*. *auris* exhibited a much lower virulence than *C*. *albicans* in both mouse and *G*. *mellonella* models (Fig. 7 and S1). However, in the *G*. *mellonella* model, *C*. *auris* was much more virulent than *C*. *glabrata* in terms of survival rates (Figure S1). These results are largely consistent with previous reports that also showed that *C*. *albicans* was much more virulent than *C*. *auris*^19,22^. The discrepancy between the Fakhim study^22^ and ours in fungal burden assays could be due to that the different mouse strains and *Candida* isolates were used. *C*. *albicans* and *C*. *auris* have comparative Sap activities at both 25 °C and 37 °C. However, the two species differ in their filamentous growth ability, which may contribute to their different abilities to cause infections.

## Materials and methods

### Strains and culture conditions

*Candida auris, Candida albicans*, and *Candida glabrata* strains were routinely grown in YPD medium (20 g/L peptone, 10 g/L yeast extract, 20 g/L glucose; for solid medium, 20 g/L agar was added). YPD, YPD + 10% NaCl, Spider^23^, agar (2%) + serum, and modified Lee’s glucose and Lee’s GlcNAc media^24,25^ were used for the morphological assays. Solid media were supplemented with 5 μg/mL phloxine B. For the morphological assays, approximately 1 × 10^5^ cells of *C*. *auris* were spotted on the different media and cultured at 25 °C, 37 °C, or 40 °C for five days. YPD medium containing serial concentrations of copper sulfate (CuSO_4_) was used for the CuSO_4_ inhibition assays. The *C*. *auris* strain was adjusted to 5 × 10^8^ cells/mL, and then 10-fold serial dilutions of cells (2 μL) were spotted onto plates containing different media.

### Phylogenetic analysis

The internal transcribed sequences (ITS) of *C*. *auris* BJCA001 and previously reported isolates were aligned using mafft v7.015b^26^. The Maximum- Likelihood phylogenetic tree was generated using RAxML v7.3.2^27^. The General Time Reversible (GTR) model and Gamma distribution with Invariant sites (G + I) were adopted. *Schizosaccharomyces pombe* strain ATCC 38366 was used as an outgroup, whereas *Candida pseudohaemulonii*_CBS10099^T^, *Candida duobushaemulonii*_CBS7798^T^, and *C*. *haemulonis*_CBS5149^T^ served as comparators. To better illustrate the phylogenetic position of *C*. *auris*, several other *Candida* species were also included. The ITS sequences of the reported strains were acquired from the GenBank (https://www.ncbi.nlm.nih.gov/) or CGD (http://www.candidagenome.org/) databases directly or extracted from the genome sequences^3^.

### Minimal inhibitory concentration (MIC) assay

Antifungal susceptibility testing was performed using the Sensititre YeastOne^TM^ methodology (Thermo Scientific, Inc., Cleveland, OH, USA) in accordance with the manufacturer’s instructions. MICs were determined after 24 h of incubation at 35 °C. *Candida krusei* ATCC 6258 and *Candida parapsilosis* ATCC 22019 were used as quality controls.

### Microscopy assay

Cells grown on nutrient agar were collected and used for morphological analysis. Differential interference contrast (DIC) optics was used for standard cellular morphology assays. The 4’, 6-diamidino-2-phenylindole (DAPI, Sigma-Aldrich, Inc., Beijing) was used for nuclear staining, and Calcofluor white (Sigma-Aldrich, Inc., Beijing) was used for septa/chitin staining as described previously^28^. Scanning electron microscopy (SEM) assays were performed as described in our previous publication^29^. The cell growth conditions are described in the figure legends.

### Secreted aspartyl proteinase (Sap) activity assay

Sap activity was tested using the YCB-BSA method as described previously^30^. Briefly, cells of *C*. *auris* or *C*. *albicans* were initially grown on YPD medium at 30 °C for 24 h. Next, 5 × 10^6^ cells of each strain in 5 μL ddH_2_O were spotted onto YCB-BSA plates and cultured at 25 °C, 37 °C, 40 °C, or 42 °C for five days. The width of the BSA precipitation rings (halos), reflecting the activity of Saps, was examined on the fifth day. Three biological repeats were performed.

### Mouse systemic infection models

All the animal experiments were performed according to the guidelines approved by the Animal Care and Use Committee of the Institute of Microbiology, Chinese Academy of Sciences (approval number: SQIMCAS2018009). Mouse systemic infection experiments were performed as described in our previous reports with slight modifications^30^. Five-week-old female BALB/c mice were used in this study. For the survival rate test, 10 mice were used for infection of each strain. 1 × 10^6^ cells of *C*. *albicans* or 1 × 10^7^ cells of *C*. *auris* in 250 μL 1 × PBS were injected into each mouse via the lateral tail vein.

For fungal burden assays, five mice were used for each intravenous infection of *C*. *auris* or *C*. *albicans*. The mice were humanely killed by cervical dislocation at 24 h post-infection. The brain, kidney, spleen, liver, and lung of each infected mouse were removed, weighed, and homogenized for colony-forming unit (CFU) analysis on YPD medium.

### Galleria mellonella infection model

*Galleria mellonella* in the final instar larval stage were purchased from Tianjin Huiyu biological technology Co. LTD. (Tianjin, China). Larvae with a similar size (0.3~0.4 g) were used for infection assays. Cells of *C*. *auris, C*. *albicans, and C*. *glabrata* were cultured on YPD medium at 30 °C for 24 h. Cells were then collected and washed twice with 1 × PBS, and 5 × 10^6^, 1 × 10^6^, or 5 × 10^5^ cells in 10 µL 1 × PBS were injected into each larva using a syringe as described previously^31^. After injection, the larvae were placed in plastic culture dishes and incubated at 30 °C in the dark.

## Electronic supplementary material

Figure S1
