# Supplementary figures and images for: The first isolate of Candida auris in China: clinical and biological aspects
Source: Emerg Microbes Infect. 2018 May 18;7:93. doi: 10.1038/s41426-018-0095-0 (PMC5959928; doi:10.1038/s41426-018-0095-0)

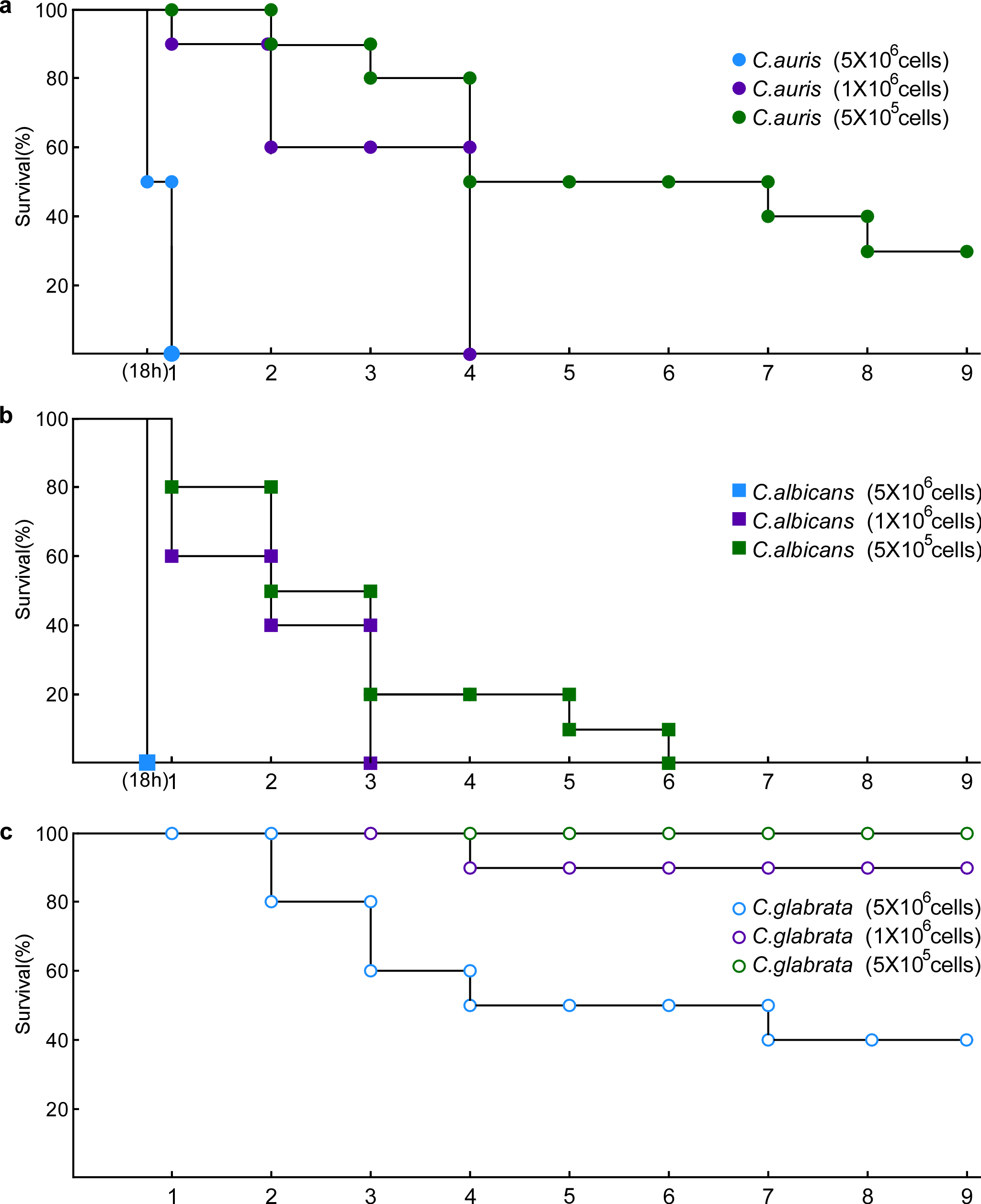

Supplement: Supplementary file 1 — Figure S1 [file 41426_2018_95_MOESM1_ESM.tif]
